# A comparison of laryngeal mask airway-supreme and endotracheal tube use with respect to airway protection in patients undergoing septoplasty: a randomized, single-blind, controlled clinical trial

**DOI:** 10.1186/s12871-020-01222-4

**Published:** 2021-01-07

**Authors:** Erol Karaaslan, Sedat Akbas, Ahmet Selim Ozkan, Cemil Colak, Zekine Begec

**Affiliations:** 1 Department of Anesthesiology and Reanimation, Inonu University Medical Faculty, Malatya, Turkey; 2 Department of Biostatistics and Medical Informatics, Inonu University Medical Faculty, Malatya, Turkey

**Keywords:** Airway protection, LMA supreme, Septoplasty, Fiberoptic bronchoscopy, Sore throat

## Abstract

**Background:**

There are doubts among anesthesiologists on the use of the Laryngeal Mask Airway (LMA) in nasal surgeries because of concerns about the occurrence of blood leakages to the airway. We hypothesized that the use of LMA-Supreme (LMA-S) in nasal surgery is comparable with endotracheal tube (ETT) according to airway protection against blood leakage through the fiberoptic bronchoscopy, oropharyngeal leakage pressure (OLP), heart rate (HR), mean arterial pressure (MAP), and postoperative adverse events.

**Methods:**

The present study was conducted in a prospective, randomized, single-blind, controlled manner on 80 patients, who underwent septoplasty procedures under general anesthesia, after dividing them randomly into two groups according to the device used (LMA-S or ETT). The presence of blood in the airway (glottis/trachea, distal trachea) was analyzed with the fiberoptic bronchoscope and a four-point scale. Both groups were evaluated for OLP; HR; MAP; postoperative sore throat, nausea, and vomiting; dysphagia; and dysphonia.

**Results:**

In the fiberoptic evaluation of the airway postoperatively, less blood leakage was detected in both anatomic areas in the LMA-S group than in the ETT group (glottis/trachea, *p* = 0.004; distal trachea, *p* = 0.034). Sore throat was detected less frequently in the LMA-S group at a significant level in the 2nd, 6th, and 12th hours of postoperative period; however, other adverse events were similar in both groups. Hemodynamic parameters were not different between the two groups.

**Conclusion:**

The present findings demonstrate that the LMA-S provided more effective airway protection than the ETT in preventing blood leakage in the septoplasty procedures. We believe that the LMA-S can be used safely and as an alternative to the ETT in septoplasty cases.

**Trial registration:**

This trial is registered at the US National Institutes of Health (ClinicalTrials.gov) # NCT03903679 on April 5, 2019.

## Background

For many years, the endotracheal tube (ETT) has been accepted as the standard method for airway safety [1]. High pressure use of cuffed tubes may lead to mucosal hypoperfusion and injury [2]. The Laryngeal Mask Airway (LMA), which was first produced in the 1980s, has been used as a minimally invasive airway device in many general anesthesia practices and has become an alternative to the ETT in many surgeries [3–5].

The LMA has many advantages over the ETT, such as having no direct contact with the tracheal mucosa, no need for direct laryngoscopy during inserting, and less adverse events such as lower frequency of coughing and decreased oxygen saturation during emergence, and lower incidence of sore throat in adults [6]. In recent years, new generation SADs have been developed, such as LMA-Supreme™, which is more superior than LMA-Classic with respect to safety of airway management [7].

The LMA-S is one of the second-generation semi-rigid and elliptical LMAs; it can be easily and quickly inserted without placing fingers in the patient’s mouth, and it does not include latex. These second-generation models were designed to provide higher sealing pressures than the LMA-Classic. In addition, they have a duct for facilitating early identification of regurgitation prior to aspiration and also helping gastric decompression [8]. There are hesitations among anesthesiologists about using the LMA because of concerns regarding vocal cord contamination and tracheal blood leakage in nasal surgical procedures [9]. There are studies on nasal surgeries with LMA-Classic in the literature, but studies on second-generation LMAs, which protect the airway better than classical LMAs, are limited.

Blood leaking from the nasopharynx to the hypopharynx in nasal surgeries contaminates the vocal folds and the tracheobronchial tree. We hypothesized that the LMA-S will reduce the blood leakage to the glottis/trachea and distal trachea. The primary outcome of this study, which was conducted on septoplasty cases, was to compare the effectiveness of the LMA-S and the ETT in protecting the airway against blood leakage using fiberoptic bronchoscopy. The secondary outcome of this study was to evaluate oropharyngeal leakage pressure (OLP); hemodynamic response, including HR and MAP; and postoperative adverse events.

## Methods

### Protocol

This study was approved by the Local Ethics Committee (Protocol no: 2018/165) and registered at www.ClinicalTrials.gov (# NCT03903679). We conducted a prospective, randomized, single-blind, and controlled clinical study with 80 adult patients undergoing septoplasty surgery at Inonu University Hospital, Malatya, Turkey. This study was prepared in accordance with the Consolidated Standards of Reporting Trials (CONSORT) [10].

### Study design

This study was planned as a randomized prospective study. Patients were randomly assigned to either LMA-S (*n* = 40) or ETT (n = 40) group; randomization (1:1) was based on a computer generated random numbers table, using MedCalc v. 16 statistical software for Windows (medcalc.com.tr). The patients who agreed to participate voluntarily were told about the features of both airway devices to be used in airway management. The patient wasn’t told which device was to be used. Patients was blinded by this method. At the same time, the nurses who had the task of evaluation in the study in PACU and the related service were blinded by not being informed about the airway device used in the operation.

### Study participants

The patients agreed to participate in the study voluntarily. After they were informed about the general information of the study, they completed the written informed consent forms. The study was conducted with patients, aged 18–65, who were scheduled for elective nasal septum surgery (American Society of Anesthesiologists (ASA) I-II).

In the preoperative anesthesia evaluation, patients with scores higher than ASA II and patients who had severe respiratory, hepatic, or renal dysfunction; neurology and/or psychiatry disorders; an allergy to anesthesia drugs, a body mass index (BMI) over 30; predictors of a difficult intubation (cervical spine pathology, modified Mallampati class 4, or thyromental distance < 65 mm); or a history of gastroesophageal reflux or hiatal hernia were excluded from the study.

### Preoperative procedures

The general anesthesia was standardized for all patients. Standard monitoring consisting of noninvasive blood pressure (NIBP), pulse oximetry (SpO2), electrocardiogram (ECG) were applied to the patients who were admitted to the surgery room. Following this stage, preoperative oxygenation was performed with 100% oxygen for 3 min (min).

### General anesthesia

Anesthesia induction was carried out with propofol 2.5 mg.kg^− 1^ intravenously (IV) + remifentanil 3 μg.kg^− 1^ IV in both groups, and no myorelaxants were used. After the patients lost consciousness and following adequate mandibular relaxation, the LMA-S was inserted using a single-hand rotation technique with the cuff lubricated. The back side of the cuff was lubricated with a water-soluble gel (K-Y®, Johnson & Johnson™, Les Moulineaux, France), and the cuff of the mask was fully deflated before insertion. Airway management was performed by the same anesthesiologist, who had at least 5 years of experience. Cases in which the airway devices was not performed successfully after the second trial were excluded from the study.

The LMA-S size was chosen according to body weight in accordance with the manufacturer guidelines (< 50 kg, size 3; 50–70 kg, size 4; 70–100 kg, size 5). After the insertion of LMA-S, if a leak sound occurs with gentle manual ventilation, a larger sized LMA-S was used which is in accordance with daily clinical practice [11, 12].

Cuffed endotracheal tubes with internal diameters of 7 mm and 8 mm were used for female and male patients, respectively. The LMA-S intracuff pressure of 60 cm H_2_O was adjusted to the ETT cuff pressure of 20 cm H_2_O/1 manometer (Portex Cufator Endotracheal Tube Inflator and Manometer, Portex® Limited, Hythe, Kent, United Kingdom) [13]. Successful airway management was confirmed by the lack of sound from leaking air from the mouth, the expansion of the chest during ventilation, five consecutive capnography curves on the monitor, and auscultation. With the LMA-S, a nasogastric tube was inserted into the LMA drainage tube. Air leakage to the stomach was controlled by checking for bubbles (foam) at the proximal end of the nasogastric tube [14].

After successful airway management, volume-controlled mechanical ventilation (Dräger Primus ventilator, Dräger AG, Lübeck, Germany) was initiated at a tidal volume of 8 ml/kg and a respiratory rate of 12 breaths/min; then, the respiratory rate was adjusted to maintain an EtCO_2_ concentration of 35–45 mmHg. Anesthesia was maintained in all patients with sevoflurane mixture of FiO2 0.5 and air. The depth of anesthesia was monitored using the Bispectral Index (BIS, VISTA Monitoring System, Massachusetts, United States of America). BIS sensors were placed in the right and left frontal areas under the hairline and covered with tape to prevent exposure to light. MAC value of Sevoflurane was titrated so that the BIS value was between 40 and 60 during operation. In addition, 0.1–0.3 μg/kg/min IV remifentanil infusion was used.

### Outcome measures

The primary outcome of the present study was to evaluate tracheal blood leakage in patients, who underwent septoplasty and whose airway patency was maintained by the LMA-S or the ETT, using a fiberoptic bronchoscope. At the end of the surgeries, a 3.5 mm fiberscope (Karl Storz GmbH & Co. KG, Tuttlingen, Germany) was used to examine blood leakage through both airway devices (glottis/trachea, distal trachea). In the patients for whom an ETT was used, the posterior oropharynx was aspirated carefully at the end of the surgeries; after extubation, the presence of blood around the distal area of the ETT cuff was examined. To evaluate the presence of blood (glottis/trachea, distal trachea), a four-point scale was used (1 = absent, 2 = mild, 3 = moderate, 4 = severe) [9]. Blood leakage was evaluated by another anesthesiologist experienced with fiberoptic bronchoscopy.

The secondary outcome of the study was to evaluate OLP; hemodynamic response, including HR and MAP; adverse events, including sore throat, nausea, and vomiting; dysphagia; and dysphonia. OLPs were measured for the LMA-S and the ETT when the head was in a neutral position. The O_2_ flow was set to 3 L/min in the flowmeter, and the expiratory valve was closed. To prevent bias, one researcher covered the airway device so that the airway device type was not visible; then, another researcher checked the peak pressure value as soon as the first researcher heard an oropharyngeal leak sound, confirming that the pressure remained constant (manometer stability test) [15]. This value was recorded as the OLP. To avoid exposing the lungs to barotrauma, when the peak inspiratory pressure reached 40 cm H_2_O, the expiratory valve was opened, and the test was ended. MAP and HR were measured immediately before anesthesia induction and at the 5th, 15th, 30th min of perioperatively and 5th min after the extubation and airway placement were confirmed.

In the 24-h postoperative period, sore throat, nausea, vomiting, dysphagia, and dysphonia were recorded at the 2nd, 6th, 12th, and 24th hours. Sore throat was defined as continuous pain felt independently from swallowing, evaluated using a numeric rating scale (NRS) between 0 and 10. According to the NRS, the sore throat score was evaluated as “0-1 none; 2-4 mild; 5-7 moderate; and 8-10 severe” [16]. A 4-point scale was used to determine the severity of the nausea and vomiting (0 = no nausea, 1 = mild-moderate nausea, 2 = less than two vomits an hour, 3 = more than two vomits an hour). Dysphonia was defined as difficulty in speaking because of difficult speech or pain. Dysphagia was defined as difficulty in swallowing or painful swallowing [17].

### Postoperative management

The patients who opened their eyes with stimuli and had regular spontaneous breathing, a respiratory rate of 12–20/min, and an oxygen saturation larger than 95% were extubated and taken to the recovery room. The patients were transferred to the Otorhinolaryngology ward when they achieved a modified Aldrete’s score of nine or greater (on a 0–12 scale), indicating recovery sufficient for the patient to be transferred from Postoperative Care Unit (PACU) to the ward [18]. All patients received a standard postoperative analgesic regime of paracetamol (1 g) and tramadol (1–2 mg/kg) IV.

### Sample size

A type I error (alpha) 0.05, strength of the test (1-beta) 0.9, effect size 0.71 for the amount of blood in the primary output variable airway (glottis/trachea), and the alternative hypothesis (H1) were employed to calculate the minimum sample size. Subsequently, the minimum sample size was determined to be 40 for each group and 80 patients in total in order to find a significant difference [9]. The sample size was calculated by a web-based software (http://biostatapps.inonu.edu.tr/WSSPAS/).

### Statistical analysis

Quantitative data were expressed as mean with standard deviation and median (min-max) values depending on the variable distribution, and qualitative data were summarized as frequency (percentage) for the related variables. Normality distribution was assessed using the Shapiro Wilk test. Quantitative data was analyzed using the independent samples t-test and the Mann Whitney U-test, where appropriate. Qualitative data was analyzed with the Pearson chi-square or the Fisher’s exact test, where appropriate. A value of *P* < 0.05 was considered as significant. IBM SPSS Statistics version 25.0 for Windows was used for the statistical analysis.

## Results

**Fig. 1:**
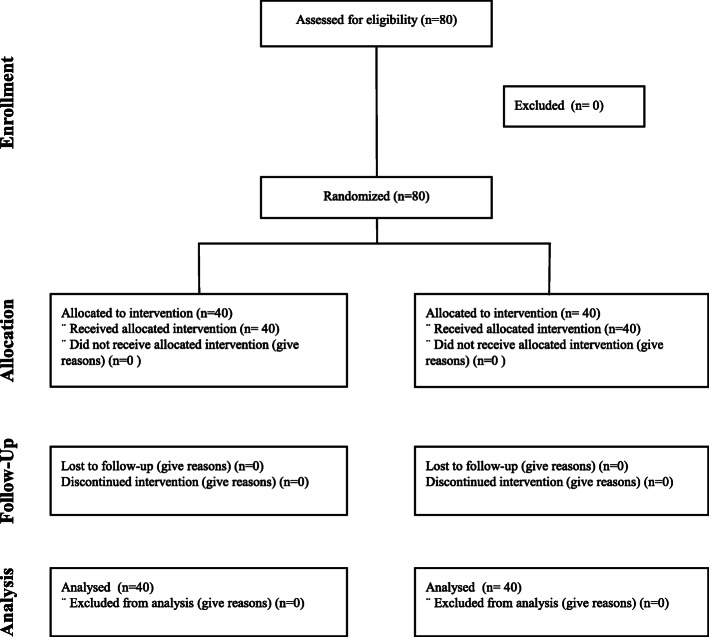
Flow Diagram. CONSORT flow chart for patients’ recruitment

A total of 80 patients were included in this study. A flow diagram is presented in Fig. 1. The age, weight, height, gender, Mallampati score, BMI, and ASA values ​​were similar in both groups. Duration of surgery was 54.03 ± 13.75 min and 51.48 ± 11.92 min (p: 0.613) in LMA-S and ETT Groups, respectively. Duration of anesthesia was 70.95 ± 13.55 min and 69.53 ± 11.42 min (p: 0.378) in LMA-S and ETT Groups, respectively, and did not differ between groups. The rates of success for the first insertion attempt were not significantly different between the two groups (36 (90%) in the LMA-S group and 34 (85%) in the ETT group). For all patients with an unsuccessful first insertion attempt, success was achieved in the second attempt. The demographic characteristics of the patients are given in Table 1.

**Table 1:** Characteristics of the Patients

	LMA-S (***n*** = 40)		ETT (***n*** = 40)		
Variable	Range	Mean ± SD or n(%)	Range	Mean ± SD or n(%)	***p*** value
Age, years	18–65	34.18 ± 14.16	18–64	35.78 ± 13.59	0.608^a^
Gender, male/female	–	21/19	–	19/21	0.655^b^
Height, cm	150–183	168.55 ± 8.22	152–185	167.63 ± 9.43	0.642^a^
Weight, kg	48–90	67.28 ± 9.01	47–105	69.63 ± 14.60	0.855^a^
BMI, (kg/m^2^)	19–29	23.58 ± 2.41	18–31	24.13 ± 3.27	0.323^a^
ASA, n					
1	–	23 (57.5%)	–	21 (52.5%)	0.653^b^
2	–	17 (42.5%)	–	19 (47.5%)	
Mallampati Score					
1	–	25 (62.5%)	–	26 (65.0%)	0.816^b^
2	–	15 (37.5%)	–	14 (35%)	
Smoking, n (%)	–	12 (30%)	–	10 (25%)	0.802^b^

*ASA* American Society of Anesthesiology; *BMI* Body Mass Index; *cm* centimeter; *kg* kilogram; *min* minutes; *n* number, *SD* Standard Deviation; a: independent samples t test; b: Chi-square test

The blood leakage in the glottis/trachea was significantly lower in the LMA-S group in the ETT group (*p* = 0.004). The blood leakage in the distal trachea was significantly lower in the LMA-S group than in the ETT group (*p* = 0.034). OLP was 21.60 ± 3.74 cmH_2_O in the LMA-S group and 22.88 ± 5.52 cmH_2_O in the ETT group; there was no statistically significant difference between the groups (*p* = 0.577). Clinical outcome variables by airway type are presented in Table 2.

**Table 2:**
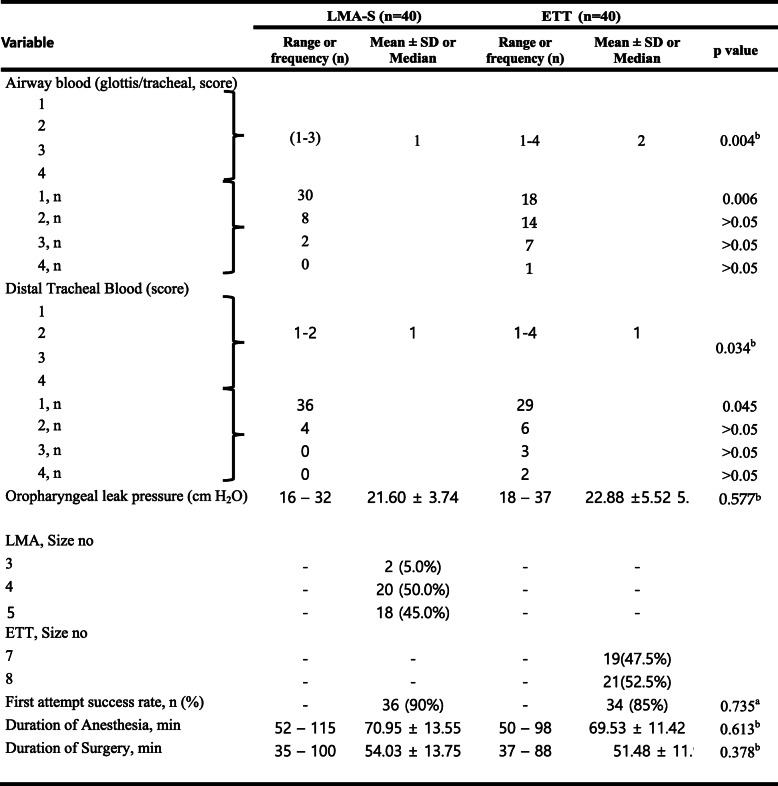
Clinical outcomes variables by airway type

Airway blood (glottis/tracheal and distal tracheal) scores: 1 = none, 2 = mild, 3 = moderate, 4 = severe; a: Chi-square test, b: independent samples t test. *LMA-S* Laryngeal Mask Airway Supreme, *ETT* endotracheal tube

Heart rate was significantly lower in the LMA-S group at 15 min after intubation than in the ETT group (*p* = 0.003). There was no significant difference between the groups at other time points. The HR of the patients in the two groups at various time points are presented in Table 3. MAP was significantly lower in the LMA-S group at 15 min after intubation than in the ETT group (*p* = 0.029). There was no significant difference between the groups at other time points. The MAP of the patients in the two groups at various time points are presented in Table 4. Sore throat was significantly higher in the ETT group at the 2nd, 6th, and 12th postoperative hours (*p* = 0.003, *p* = 0.017 and *p* < 0.001, respectively).

**Table 3:** Heart rate of patients at various time points in two groups

Variable	LMA-S (***n*** = 40)		ETT (***n*** = 40)		
	Range	Mean ± SD	Range	Mean ± SD	***p*** value
**Baseline**	60–115	84.50 ± 14.65	58–121	82.78 ± 14.07	0,647^a^
**After intubation**					
5th min	52–98	75.20 ± 12.07	54–119	80,30 ± 15.20	0.127^a^
15th min	51–90	70.58 ± 11.16	53–116	81.15 ± 16.13	0.003^a^
30th min	57–96	74.43 ± 9.54	60–110	77.93 ± 12.13	0.264^a^
**After extubation**					
5th min	56–104	77.25 ± 10.60	63–103	77.73 ± 9.98	0.878^a^

a: independent samples t test;

**Table 4:** Mean arterial pressure of patients at various timepoints in two groups

Variable	LMA-S (***n*** = 40)		ETT (***n*** = 40)		
	Range	Mean ± SD	Range	Mean ± SD	***p*** value
**Baseline**	60–115	89.88 ± 13.59	57–114	84.23 ± 16.18	0.083^a^
**After intubation**					
5th min	43–94	70.63 ± 11.16	46–118	74.30 ± 16.06	0.438^a^
15th min	57–121	71.7 ± 14.78	57–121	79.03 ± 15.04	0.029^a^
30th min	49–98	71.28 ± 11.39	47–112	75.25 ± 13.38	0.260^a^
**After extubation**					
5th min	56–95	74.53 ± 9.80	56–105	75.00 ± 72.50	0.988^a^

a: independent samples t test;

There were no differences between the groups in terms of having a sore throat at the 24th postoperative hour (*p* = 0.057). Nausea, vomiting, dysphagia, and dysphonia were similar in both groups. The postoperative adverse events are presented in Table 5.

**Table 5:** Postoperative adverse events

	LMA-S (***n*** = 40)				ETT (***n*** = 40)							
Variable	n (%)				n (%)				***p*** values			
Postoperative period	2nd hour	6th hour	12th hour	24th hour	2nd hour	6th hour	12th hour	24th hour	P_**2nd**_^**a**^	P_**6th**_^**a**^	P_**12th**_^**a**^	P_**24h**_^**a**^
**Sore throat** (None/mild/moderate/severity)	28/8/4/0 (70/20/10/0)	28/11/1/0 (70/28/2/0)	37/3/0/0 (92.5/7.5/0/0)	39/1/0/0 (97.5/2.5/0/0)	15/8/14/3 (37.5/20/35/7.59	16/17/6/1 (40/42.5/15/2.5)	22/14/4/0 (55/35/10/0)	39/1/0/0 (97.5/2.5/0/0)	**0.003**	**0.017**	**< 0.001**	0.057
**Nausea-vomiting** (0/1/2/3)^a^	28/7/5/0 (70/17.5/12.5/0)	35/3/1/1 887.5/7.5/2.5/2.5)	38/1/1/0 (95/2.5/2.5/0)	39/1/0/0 (97.5/2.5/0/0)	29/4/6/1 (72.5/10/15/2.5)	36/0/4/0 (90/0/10/0)	39/1/0/0 (97.5/2.5/0/0)	40/0/0/0 (100/0/0/0)	0.76	0.12	0.61	1
**Dysphagia** (Absent/present)	35/5 (87.5/12.59)	39/1 (97.5/2.59)	40/0 (100/0)	40/0 (100/0)	33/7 (82.5/17.5)	34/6 (85/15)	37/3 (92.5/7.5)	37/3 (92.5/7.5)	0.75	0.10	0.24	0.24
**Dysphonia** (Absent/present)	38/2 (95/5)	37/3 (92.5/7.5)	38/2 (95/5)	39/1 (97.5/2.5)	37/3 (92.5/7.5)	37/3 (92.5/7.5)	38/2 (95/5)	39/1 (97.5/2.59)	1	1	1	1

^a^Nausea and vomiting score: 0 = none, 1 = mild-moderate nausea, 2 = vomiting less than 2 times per hour, 3 = vomiting more than 2 times per hour; a: Pearson chi-square test

## Discussion

In this study, in which LMA-S and ETT use was compared in terms of tracheal blood leakage and adverse events in septoplasty cases, the blood leakage in the LMA-S group was statistically less than in the ETT group; this was determined by examining the airway for the presence of blood with a fiberoptic bronchoscope.

Many anesthetists and otolaryngologists are concerned about using the LMA due to concerns that blood leakage from the posterior to the hypopharynx during nasal and endoscopic sinus surgery could contaminate the vocal folds and the tracheobronchial tree, causing laryngospasm and bronchospasm. Therefore, for such operations, the ETT has been the first choice for airway safety. In the literature, there are studies comparing LMA and ETT use in nasal surgeries [9, 19]. Al-Mazrou et al. reported that LMA is a suitable method for paediatric patients undergoing sinonasal surgery [19]. However, a standardization of the cases could not be made in most studies. We achieved surgical standardization by conducting our study on septoplasty cases only. In addition, the presence of blood in the airway devices was evaluated visually, and indirect data was obtained about tracheal leakage. In our study, the presence of blood in the airway (glottis/trachea, distal trachea) was evaluated more objectively using a fiberoptic bronchoscope.

In septoplasty cases, the LMA-S avoids blood leakage to the glottis and the trachea, with its high sealing effect in the oropharynx, by surrounding the supraglottic and glottic area [9]. With the ETT, however, as the cuff is below the glottis level, blood produced during surgery can easily reach the glottis and lower parts of the airway using the outer surface of the ETT [20]. Although the ETT is considered to protect the airway better in terms of blood leakage through its traditional approach, in our study, we hypothesized that the LMA-S, which is a minimally invasive airway device with a high sealing pressure, would reduce blood leakage to the glottis-distal trachea line.

Kaplan et al. conducted a study in which 74 patients, who underwent septoplasty or endoscopic sinus surgery, were compared in terms of ETT and LMA-Classic use; they reported that the presence of blood in the glottis/trachea level was at a less significant level in the LMA-Classic group than in the ETT group [9]. However, they also reported that there was blood in fewer cases in the ETT group (3.2%) than the CLMA group (19.6%), although it was not statistically significant in the distal trachea. Kaplan et al. also reported that the ETT protected the distal trachea better. Williams et al. conducted a study with children and adult tonsillectomy cases using the LMA-Classic and the ETT; at the end of surgery, they observed less blood in the larynx and trachea in the LMA-Classic group when an evaluation was made using a fiberoptic bronchoscope [21]. This is in agreement with the results of our study. Ahmet et al. compared ETT and reinforced LMA use in 200 septal, sinus, and septal and sinus (combined) surgery patients and reported that the blood contamination was less in the LMA cuff in visual evaluation [20]. In the present study, blood leakage in the glottis/trachea and distal trachea occurred less frequently in the LMA-S (glottis/trachea: LMA-S: 10 cases (25%), ETT: 22 cases (45%), *p* = 0.004); distal trachea: LMA-S: 4 cases (10%), ETT: 11 cases (27.5%), *p* = 0.034). Unlike Kaplan et al., we found significantly less blood in both the glottis/trachea and the distal trachea in the LMA-S group. The reasons for the differences between the two results may be, first that our study examined only septoplasty cases, while Kaplan et al. included septoplasty, endoscopic sinus surgery, and combined septoplasty/endoscopic sinus surgery cases in their study. Second, in our study, the intracuff pressure was applied as 60 mm H_2_O in the LMA-S and 20 H_2_O in the ETT, but Kaplan et al. did not report any information on which intracuff pressures they used in the airway devices. Therefore, differences in cuff pressures may have affected the results. In many studies in which the LMA and the ETT were compared without using fiberoptic bronchoscopes in patients who underwent nasal and sinus surgeries, it was concluded that the LMA provided better airway protection [22]. Therefore, evaluating blood in the airway with fiberoptic bronchoscope is more objective approach than visually indirectly evaluating the presence of blood on the cuff. In summary, we showed that the LMA-S provided better protection than the ETT in both anatomical regions of the airway.

Oropharyngeal leakage pressure is a gas leak occurring around the airway device. In general, the verification of the position of the airway device shows the success of positive-pressure ventilation and the degree of airway protection. It is also used to evaluate the effectiveness of different airway devices [23]. Seet et al. reported that OLP was 21 cmH_2_O in 99 cases in which the LMA-S was used with an intracuff pressure of 60 cmH_2_O [24]. In another study, which was conducted with the LMA-S at three different intracuff pressures (80, 60, 40 H_2_O) in 123 cases, OLP was measured as 26, 20, and 18 H_2_O, respectively [25]. In our study, the OLP value was measured as 21.60 cmH_2_O in LMA-S and 22.8 in ETT, and did not differ between the groups. In summary, the OLP values in these other studies are consistent with our OLP values.

Postoperative sore throat is among the pharyngolaryngeal adverse events that commonly occur after general anesthesia, decreasing patient satisfaction and causing prolonged hospital stays. In addition to the direct trauma of the rigid materials that are inserted in the upper airway, the physical tension caused by the laryngoscopy, tube size, gender, surgery type, and cuff pressures may cause postoperative throat complaints after tracheal intubation [26, 27]. In their study, Hermite et al. compared two different supraglottic airway devices (LMA-S, and Laryngeal Mask Airway Unique (LMA-U)) with an intracuff pressure of 60 cmH_2_O; they reported no significant differences between the LMA-S and other airway devices in terms of postoperative sore throat [28]. For this reason, in our study, we used an intracuff pressure of 60 cmH_2_O as a reference. Barreriera et al. compared LMA-S and ETT use in their study and found that the incidence of sore throat was significantly higher in the ETT group using a ETT cuff pressure of 25–30 mm cmH_2_O [29]. Similarly, Radu et al. compared ETT and LMA use in breast surgery patients and found incidences of sore throat at the 6th postoperative hour to be significantly higher with the ETT [30]. Similarly, in our study, sore throat complaints were significantly lower in the LMA-S group at the 2nd, 6th, and 12th postoperative hours (*p* = 0.003, *p* = 0.017 and *p* < 0.001, respectively). Therefore, we believe that the LMA-S causes fewer sore throats at appropriate intracuff pressures in septoplasty cases than the ETT.

## Limitations

Our study had some limitations. First, in the ETT group, the presence of blood at the vocal cord level was evaluated visually by the presence of blood on the cuff after extubation, since it was not possible to assess it with a flexible bronchoscope. Secondly, the amount of bleeding in the airway could not be measured due to technical difficulties of aspiration and the risk of undesirable airway reflexes (laryngospasm, bronchospasm).

## Conclusion

As evident in our findings, we have determined that the LMA-S is superior to the ETT in protecting the airway against blood contamination and reducing postoperative sore throat associated with general anesthesia in septoplasty surgeries. We also believe that the LMA-S may be a more reliable and better alternative to the ETT for airway management in septoplasty surgeries.

## Data Availability

The datasets analyzed during the current study are available from the corresponding author on reasonable request.

## Abbreviations

LMA-S: Laryngeal Mask Airway-Supreme
ETT: Endotracheal tube
OLP: Oropharyngeal leakage pressure
HR: Heart rate
MAP: Mean arterial pressure
ASA: American Society of Anesthesiologists
BMI: Body mass index
VAS: Visual analogue scale
NRS: Numeric rating scale
